# Identification of an HLA-A2-Restricted Epitope Peptide Derived from Hypoxia-Inducible Protein 2 (HIG2)

**DOI:** 10.1371/journal.pone.0085267

**Published:** 2014-01-08

**Authors:** Sachiko Yoshimura, Takuya Tsunoda, Ryuji Osawa, Makiko Harada, Tomohisa Watanabe, Tetsuro Hikichi, Masahiro Katsuda, Motoki Miyazawa, Masaji Tani, Makoto Iwahashi, Kazuyoshi Takeda, Toyomasa Katagiri, Yusuke Nakamura, Hiroki Yamaue

**Affiliations:** 1 Second Department of Surgery, Wakayama Medical University, Wakayama, Japan; 2 OncoTherapy Science Inc, Research and Development Division, Kanagawa, Japan; 3 Laboratory of Molecular Medicine Human Genome Center, Institute of Medical Science, The University of Tokyo, Tokyo, Japan; 4 Department of Immunology, Juntendo University School of Medicine, Tokyo, Japan; 5 Division of Genome Medicine, Institute for Genome Research, The University of Tokushima, Tokushima, Japan; 6 Department of Medicine, University of Chicago, Chicago, Illinois, United States of America; University of London, St George's, United Kingdom

## Abstract

We herein report the identification of an HLA-A2 supertype-restricted epitope peptide derived from hypoxia-inducible protein 2 (HIG2), which is known to be a diagnostic marker and a potential therapeutic target for renal cell carcinoma. Among several candidate peptides predicted by the HLA-binding prediction algorithm, HIG2-9-4 peptide (VLNLYLLGV) was able to effectively induce peptide-specific cytotoxic T lymphocytes (CTLs). The established HIG2-9-4 peptide-specific CTL clone produced interferon-γ (IFN-γ) in response to HIG2-9-4 peptide-pulsed HLA-A*02:01-positive cells, as well as to cells in which HLA-A*02:01 and HIG2 were exogenously introduced. Moreover, the HIG2-9-4 peptide-specific CTL clone exerted cytotoxic activity against HIG2-expressing HLA-A*02:01-positive renal cancer cells, thus suggesting that the HIG2-9-4 peptide is naturally presented on HLA-A*02:01 of HIG-2-expressing cancer cells and is recognized by CTLs. Furthermore, we found that the HIG2-9-4 peptide could also induce CTLs under HLA-A*02:06 restriction. Taken together, these findings indicate that the HIG2-9-4 peptide is a novel HLA-A2 supertype-restricted epitope peptide that could be useful for peptide-based immunotherapy against cancer cells with HIG2 expression.

## Introduction

Renal cell carcinoma (RCC) comprises approximately 2–3% of all human malignancies [1]. Although patients with localized RCC can be curable by radical nephrectomy, approximately 30% of patients are observed to have metastasis at the time of diagnosis, and the median survival is only 1.5 years. Furthermore, 30% of patients experience a relapse after initial surgery, and no adjuvant treatment has yet been established [2]–[4]. Several molecular targeting agents, including the recently approved VEGFR tyrosine kinase inhibitor [5], were developed as novel therapeutics for RCC, but the majority of patients eventually develop treatment-resistant disease [6]–[13]. It is notable that RCC is one of the most immune responsive cancers. IL-2 based immunotherapy is currently the only curative treatment for metastatic RCC, but it is poorly tolerated, with significant side effects, and the efficacy has been limited to a 20% response rate, including a 5–10% complete response rate [14]–[17]. This limited success poses further challenges to improve the efficacy of immunotherapies for RCC. While therapeutic vaccines that induce immunity in response to tumor antigens have been under investigation for decades, the number of antigens identified in RCC and the efficacy in clinical trials have been limited [18]–[21].

Hypoxia-inducible protein 2 (*HIG2*) was first annotated as a novel gene induced by hypoxia and glucose deprivation [22]. A recent functional analysis revealed that HIG2 is a novel lipid droplet protein that stimulates intracellular lipid accumulation [23]. We reported HIG2 upregulation in RCC, and suggested its usefulness as a diagnostic biomarker for RCC [24]. Our findings also implied that HIG2 might be a good molecular target for the development of novel cancer treatment, because its expression was hardly detectable in normal organs except for the fetal kidney. Importantly, significant growth suppression of RCC cells occurred when endogenous *HIG2* was suppressed by *HIG2*-specific RNAi, suggesting that HIG2 has an essential role in the proliferation of RCC cells. An additional study revealed that HIG2 expression was found in 86% of human RCC tissue samples (80/93) and also correlated with the clinicopathological characteristics and survival of RCC patients [25].

In the present study, we focused on HIG2 as a novel tumor antigen, which induces antigen-specific cytotoxic T lymphocytes (CTLs) against RCC cells. We investigated the HIG2-derived epitope peptide restricted to HLA-A*02:01, the most common HLA class I type in Caucasians and the second most common type in the Japanese population [26], [27], and demonstrate that this epitope peptide can also be presented by another HLA-A2 supertype allele. Thus, this epitope peptide would be applicable for peptide-based immunotherapies for RCC patients with HLA-A2.

### Ethics statement

The study protocol was approved by the Institutional Review Board of OncoTherapy Science, Inc. and written informed consent was obtained from all subjects, in accordance with the guidelines of the Ethical Committee on Human Research of Wakayama Medical University, School of Medicine, OncoTherapy Science, Inc., The University of Tokyo, Juntendo University School of Medicine, The University of Tokushima and University of Chicago.

## Materials and Methods

### Peptides

HIG2-derived 9-mer and 10-mer peptides that have high binding affinity (binding score >10) to HLA-A:*02:01 were predicted by the binding prediction software “BIMAS” (http://www-bimas.cit.nih.gov/molbio/hla_bind), and the homologous sequences were examined by the homology search program “BLAST” (http://blast.ncbi.nlm.nih.gov/Blast.cgi). Selected high affinity peptides and the HLA-A*02:01-restricted HIV-derived epitope peptide (ILKEPVHGV) [28] were synthesized by Sigma (Ishikari, Japan). The purity (>90%) and the sequences of the peptides were confirmed by analytical HPLC and a mass spectrometry analysis, respectively. Peptides were dissolved in dimethylsulfoxide at 20 mg/ml and stored at −80°C.

### Cell lines

T2 (HLA-A*02:01, lymphoblast), Jiyoye (HLA-A32, Burkitt's lymphoma), EB-3 (HLA-A3/Aw32, Burkitt's lymphoma), *Cercopithecus aethiops*-derived COS7 and A498 (HLA-A*02:01, kidney carcinoma) cells were purchased from the American Type Culture Collection (Rockville, MD). PSCCA0922 (HLA-A*02:06/A*31:01, a B cell line) was provided by the Health Science Research Resources Bank (Osaka, Japan). Caki-1 (HLA-A*24:02/A*23:01, renal clear cell carcinoma) cells were provided by the Cell Resource Center for Biomedical Research Institute of Development, Aging and Cancer at Tohoku University. The HIG2 expression in A498 and Caki-1 cells was confirmed by a Western blotting analysis [24]. T2, Jiyoye, EB-3 and PSCCA0922 cells were maintained in RPMI1640 (Invitrogen, Carlsbad, CA), A498 and Caki-1 cells were maintained in EMEM (Invitrogen) and COS7 cells were maintained in DMEM (Invitrogen). Each medium was supplemented with 10% fetal bovine serum (GEMINI Bio-Products, West Sacramento, CA) and 1% antibiotic solution (Sigma-Aldrich, ST. Louis, MO).

### Gene transfection

The plasmid encoding HLA-A*02:01 was a generous gift from Dr. Kawakami (Keio University, Tokyo Japan). cDNA fragments encoding HLA-A*02:06 or *HIG2* (GenBank Accession Number NM_013332) were cloned into the pcDNA3.1/myc-His vector (Invitrogen). Plasmid DNAs containing HLA-A*02:01, HLA-A*02:06 and/or *HIG2* were transfected into COS7 cells using Fugene 6 (Roche Diagnostics, Indianapolis, IN) according to the manufacturer's instructions. COS7 cells were incubated with the transfection mixture at 37°C overnight prior to use as stimulator cells. The introduction of the targeted proteins was confirmed by a Western blotting analysis.

### *In vitro* CTL induction

CD8^+^ T cells and monocyte-derived dendritic cells (DCs) were prepared from peripheral blood of healthy volunteers (either HLA-A*02:01 or HLA-A*02:06 positive) with written informed consent. Peripheral blood mononuclear cells (PBMCs) were isolated by Ficoll-Paque PLUS (GE Healthcare, Uppsala, Sweden) and CD8^+^ T cells were harvested by positive selection with a Dynal CD8 Positive Isolation Kit (Invitrogen). Monocytes were enriched from the CD8^−^ cell population by adherence to a tissue culture dish (Becton Dickinson, Franklin Lakes, NJ) and were cultured in AIM-V (Invitrogen) containing 2% heat-inactivated autologous serum (AS), 1,000 U/ml of GM-CSF (R&D Systems, Minneapolis, MN) and 1,000 U/ml of interleukin (IL)-4 (R&D Systems) on day 1. On day 4, 0.1 KE/ml of OK-432 (Chugai Pharmaceutical Co., Tokyo, Japan) was added in the culture to induce the maturation of DCs. On day 7, DCs were pulsed with 20 µg/ml of the respective synthesized peptides in the presence of 3 µg/ml of β2-microglobulin (Sigma-Aldrich, ST. Louis, MO) in AIM-V at 37°C for 4 h [29]. These peptide-pulsed DCs were then incubated with 30 µg/ml of mitomycin C (MMC) (Kyowa Hakko Kirin Co. Ltd., Tokyo, Japan) at 37°C for 30 min. Following washing out the residual peptide and MMC, DCs were cultured with autologous CD8^+^ T cells on 48 well plates (Corning, Inc., Corning, NY) (each well contained 1.5×10^4^ peptide-pulsed DCs, 3×10^5^ CD8^+^ T cells and 10 ng/ml of IL-7 (R&D Systems) in 0.5 ml of AIM-V/2% AS). Two days later, these cultures were supplemented with IL-2 (CHIRON, Emeryville, CA) (final concentration: 20 IU/ml). On days 14 and 21, T cells were further re-stimulated with the autologous peptide-pulsed DCs, which were freshly prepared every time. On day 28, the CTL activity against peptide-pulsed T2 or PSCCA0922 cells was examined by an interferon (IFN)- γ enzyme-linked immunospot (ELISPOT) assay.

### IFN-γ enzyme-linked immunospot (ELISPOT) assay

The human IFN-γ ELISPOT kit and AEC substrate set (BD Biosciences) were used to analyze the T cell response to the respective peptides. The ELISPOT assay was performed according to the manufacturer's instructions. Briefly, T2 or PSCCA0922 cells were pulsed with 20 µg/ml of the respective peptides at 37°C for 20 h, and the residual peptide that did not bind to cells was washed out to prepare peptide-pulsed cells as the stimulator cells. After removing 500 µl of supernatant from each well of *in vitro* CTL-inducing cultures, 200 µl of cell culture suspensions were harvested from each well and distributed to two new wells (100 µl each) on Multiscreen-IP 96 well plates (Millipore, Bedford, MA). The cells were co-incubated with peptide-pulsed cells (1×10^4^ cells/well) at 37°C for 20 h. HIV peptide-pulsed cells were used as a negative control. Spots were captured and analyzed by an automated ELISPOT reader, ImmunoSPOT S4 (Cellular Technology Ltd, Shaker Heights, OH) and the ImmunoSpot Professional Software package, Version 5.0 (Cellular Technology Ltd).

### CTL expanding culture

The peptide-specific CTLs harvested from ELISPOT-positive wells after *in vitro* CTL induction were expanded by a modified protocol based on the previously described methods [30], [31]. A total of 5×10^4^ CTLs was cultured with 5×10^6^ MMC-inactivated Jiyoye or EB-3 cells (30 µg/ml at 37°C for 30 min treatment) in 25 ml of AIM-V/5% AS containing 40 ng/ml of anti-CD3 monoclonal antibody (BD Biosciences, San Diego, CA) on day 0. IL-2 was added 24 h later (final concentration: 120 IU/ml), and fresh AIM-V/5% AS containing 30 IU/ml of IL-2 was provided on days 5, 8 and 11. On day 14, CTLs were harvested and the CTL activity was examined by an IFN-γ enzyme-linked immunosorbent assay (ELISA).

### Establishment of CTL clones

CTL clones were established by the limiting dilution method. Briefly, CTLs were diluted to 0.3, 1 or 3 cells per well in 96 well round bottom plates (Corning, Inc.), and were cultured with MMC-treated 1×10^4^ Jiyoye and EB-3 cells in 125 µl AIM-V containing 5% AB serum and 30 ng/ml of an anti-CD3 monoclonal antibody on day 0. IL-2 was added to each well on day 10 (final concentration: 125 IU/ml). On day 14, an IFN-γ ELISPOT assay was performed to measure the CTL activity of each clone.

### IFN-γ enzyme-linked immunosorbent assay (ELISA)

The CTL activity was examined by IFN-γ ELISA. Peptide-pulsed cells (1×10^4^ cells/well) or gene-transfected cells (5×10^4^ cells/well) were used to stimulate CTLs at several responder/stimulator ratios in 200 µl of AIM-V/5% AS on 96 well round bottom plates (Corning Inc.). After 24 h of incubation, cell-free supernatants were harvested, and the IFN-γ production was examined by an IFN-γ ELISA kit (BD Biosciences) according to the manufacturer's instructions.

### Flow cytometry

The expression of peptide-specific T cell receptors was examined on FACS-Canto II (Becton Dickinson, San Jose, CA) using PE-conjugated peptide/MHC tetramer (Medical and Biological Laboratories, Nagoya, Japan) according to the manufacturer's instructions. Briefly, *in vitro* expanded CTLs were incubated with peptide/MHC tetramer at room temperature for 10 min, and then a FITC-conjugated anti-human CD8 mAb, APC-conjugated anti-human CD3 mAb, PE-Cy7-conjugated anti-human CD4 mAb and 7-AAD (BD Biosciences) were added and incubated at 4°C for 20 min. HIV peptide (ILKEPVHGV)/HLA-A*02: 01 tetramer was used as a negative control.

### Cytotoxicity assay

The cytotoxic activity of the induced CTL clones was tested by a 4 h ^51^Cr release assay as described previously [32]. Data are presented as the means ± SD of triplicate samples. Student's t test was used to examine the significance of the data.

## Results

### CTL induction with HLA-A*02:01-binding peptides derived from HIG2

**Figure 1:**
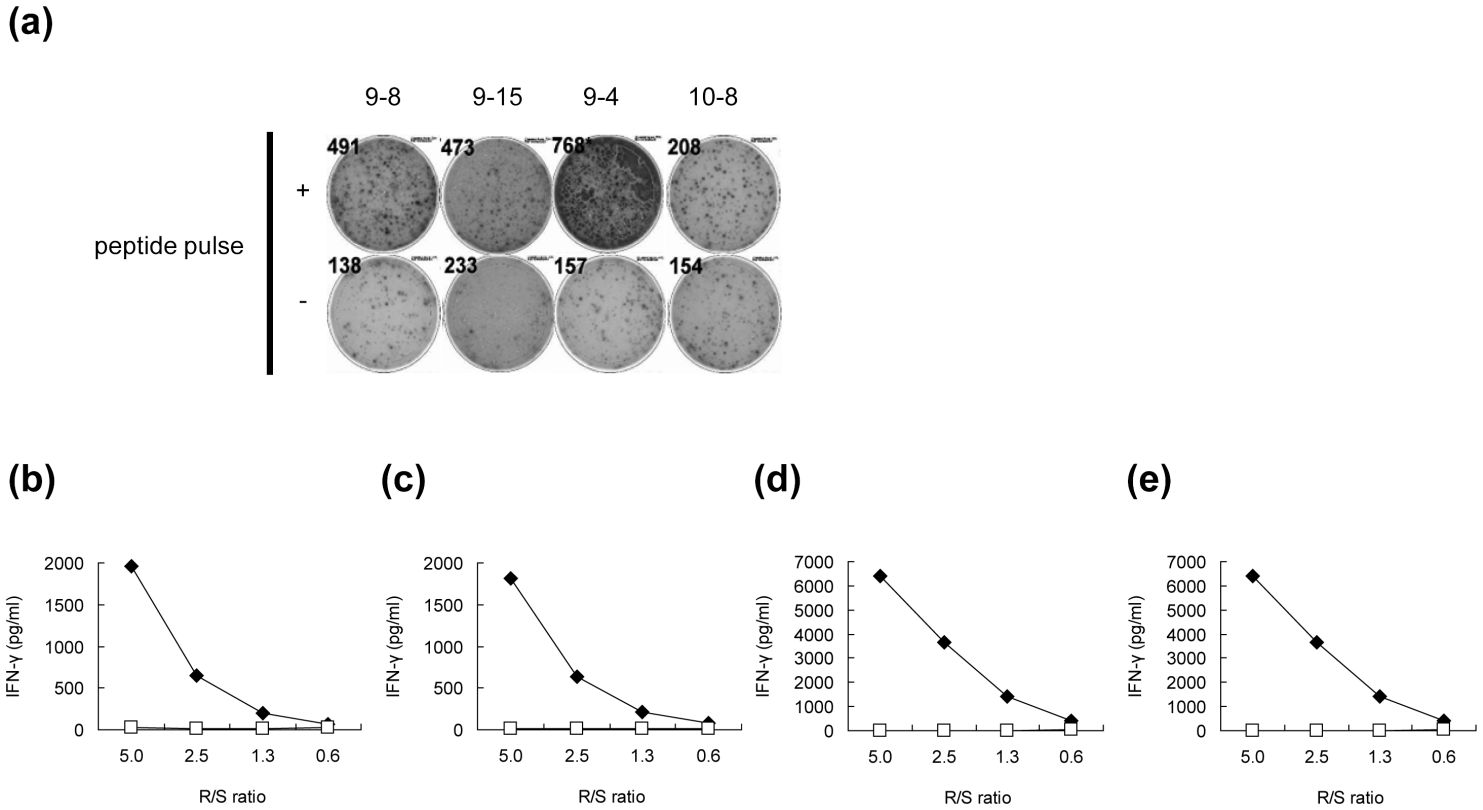
The IFN-γ production in response to the HIG2-9-8, HIG2-9-15, HIG2-9-4 or HIG2-10-8 peptide. (a) The IFN-γ production from cells induced by the indicated peptide-pulsed DCs was examined by an ELISPOT assay using T2 cells. “+” indicates the wells in which cells were stimulated with T2 cells pulsed with the indicated peptide and “−” indicates the wells in which cells were stimulated with HIV peptide-pulsed T2 cells. The IFN-γ production from cells induced with HIG2-9-8 (b), HIG2-9-15 (c), HIG2-9-4 (d) or HIG2-10-8 (e) peptide stimulation after CTL expanding culture was examined by ELISA. Cells were stimulated with T2 cells pulsed with the corresponding peptide (closed diamonds) or HIV peptide (open squares) at the indicated responder/stimulator ratio (R/S ratio). Similar results were obtained from three independent experiments.

**Figure 2:**
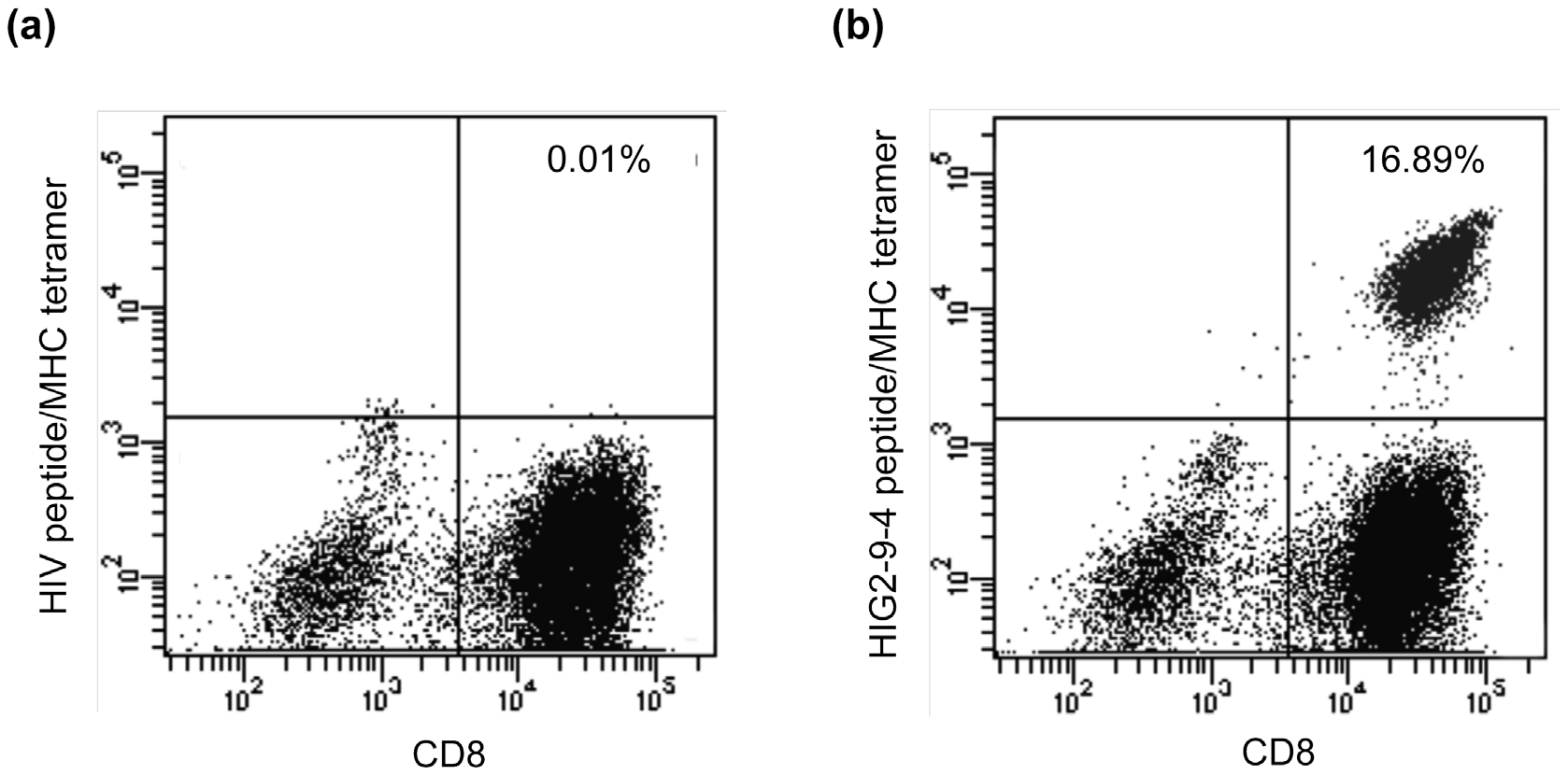
The expression of a HIG2-9-4 peptide-specific T cell receptor on CD8+ T cells. The expression of the HIG2-9-4 peptide-specific T cell receptor was examined on CD3^+^CD4^−^ cells following CTL expansion culture of HIG2-9-4 peptide-induced CTLs. (a) A quadrant gate was set based on the staining results with the HIV peptide/HLA-A*02: 01 tetramer. (b) CD8^+^ T cells expressing the HIG2-9-4 peptide/HLA-A*02: 01-specific T cell receptor were detected. Similar results were obtained from three independent experiments.

We synthesized twelve 9-mer and 10-mer peptides, corresponding to parts of the HIG2 protein that had been suggested to bind to HLA-A*02:01 by the prediction with the BIMAS program (Table 1). After *in vitro* culture to induce CTLs, IFN-γ production was observed specifically when cells were stimulated with T2 cells that had been pulsed with the HIG2-9-8 peptide (YLLGVVLTL), HIG2-9-4 peptide (VLNLYLLGV), HIG2-9-15 peptide (TLLSIFVRV) or HIG2-10-8 peptide (YLLGVVLTLL) among all of the candidate peptides shown in Table 1 (Fig. S1 showing all 12 wells of one experiment and Fig. 1a showing representative wells). After CTL-expanding culture, cells still produced IFN-γ in response to the respective peptides in a responder/stimulator ratio-dependent manner, and HIG2-9-4 peptide-specific CTLs produced a higher amount of IFN-γ than CTLs stimulated with other peptides (Figs. 1b–e). In the independent experiments using PBMCs from other 2 donors, HIG2-9-4 peptide-specific CTLs produced the highest amount of IFN-γ (data not shown). We confirmed the existence of HIG2-9-4/HLA-A*02:01-specific CD8^+^ T cells by tetramer staining. A significant population of CD3^+^CD4^−^CD8^+^ cells expressed the HIG2-9-4/HLA-A*02:01-specific T cell receptor after the expansion of cells obtained by *in vitro* CTL induction (Fig. 2).

**Table 1:** Candidate peptides derived from HIG2 restricted with HLA-A*02:01.

Peptide name	Amino acid sequence (mer)	Binding Score
HIG2-9-8	YLLGVVLTL (9)	836.253
HIG2-9-13	VLTLLSIFV (9)	650.311
HIG2-9-15	TLLSIFVRV (9)	488.951
HIG2-9-4	VLNLYLLGV (9)	271.948
HIG2-9-9	LLGVVLTLL (9)	83.527
HIG2-9-22	RVMESLEGL (9)	31.957
HIG2-9-6	NLYLLGVVL (9)	28.027
HIG2-10-8	YLLGVVLTLL (10)	836.253
HIG2-10-29	GLLESPSPGT (10)	113.047
HIG2-10-4	VLNLYLLGVV (10)	14.495
HIG2-10-15	TLLSIFVRVM (10)	13.174
HIG2-10-18	SIFVRVMESL (10)	12.248

The binding score was obtained from the BIMAS website (http://www-bimas.cit.nih.gov/molbio/hla_bind).

### Establishment of HIG2-9-4 peptide-specific CTL clones

**Figure 3:**
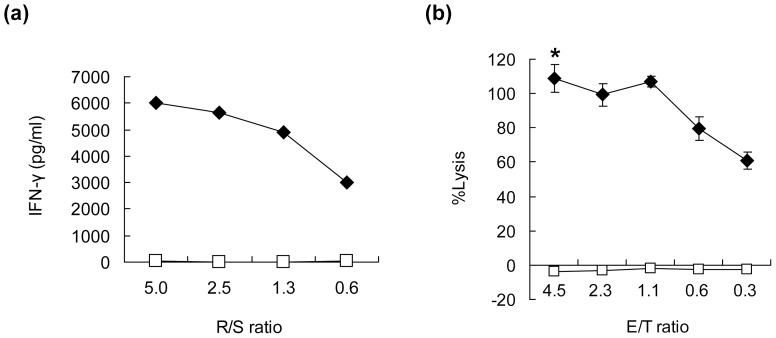
The IFN-γ production and cytotoxic activity of a HIG2-9-4 peptide-specific CTL clone. (a) An established CTL clone was stimulated with T2 cells pulsed with the HIG2-9-4 peptide (closed diamonds) or HIV peptide (open squares). The IFN-γ production in the culture supernatant was examined by ELISA. R/S ratio; responder/stimulator ratio. (b) The cytotoxic activity of the HIG2-9-4 peptide-specific CTL clone was examined against peptide-pulsed T2 cells (close diamond) or T2 cells pulsed with the HIV peptide (open square). E/T ratio; effector/target ratio. All experiments were performed in triplicate. The representative results from three independent experiments are shown. *P<0.001

We subsequently established HIG2-9-4 peptide-specific CTL clones by the limiting dilution of induced CTLs. The established HIG2-9-4 peptide-specific CTL clone produced a large amount of IFN-γ when it was stimulated with HIG2-9-4 pulsed-T2 cells, while no IFN-γ production was detected when they were stimulated with HIV-peptide-pulsed-T2 cells (Fig. 3a). Furthermore, the HIG2-9-4 peptide-specific CTL clone exerted substantial cytotoxic activity against T2 cells pulsed with the HIG2-9-4 peptide, but not those pulsed with the HIV peptide (Fig. 3b). However, we failed to establish any CTL clones that reacted with HIG2-9-8, HIG2-9-15 or HIG2-10-8 peptides, even after several attempts using multiple donors (data not shown). In addition, we found no homologous sequence to the HIG2-9-4 peptide by a homology search using the BLAST algorithm (data not shown), indicating that the HIG2-9-4 peptide is a unique epitope peptide among the candidate peptides predicted by the BIMAS program that can induce potent and stable CTLs.

### Specific CTL response to HIG2 and HLA-A*02:01-expressing cells

**Figure 4:**
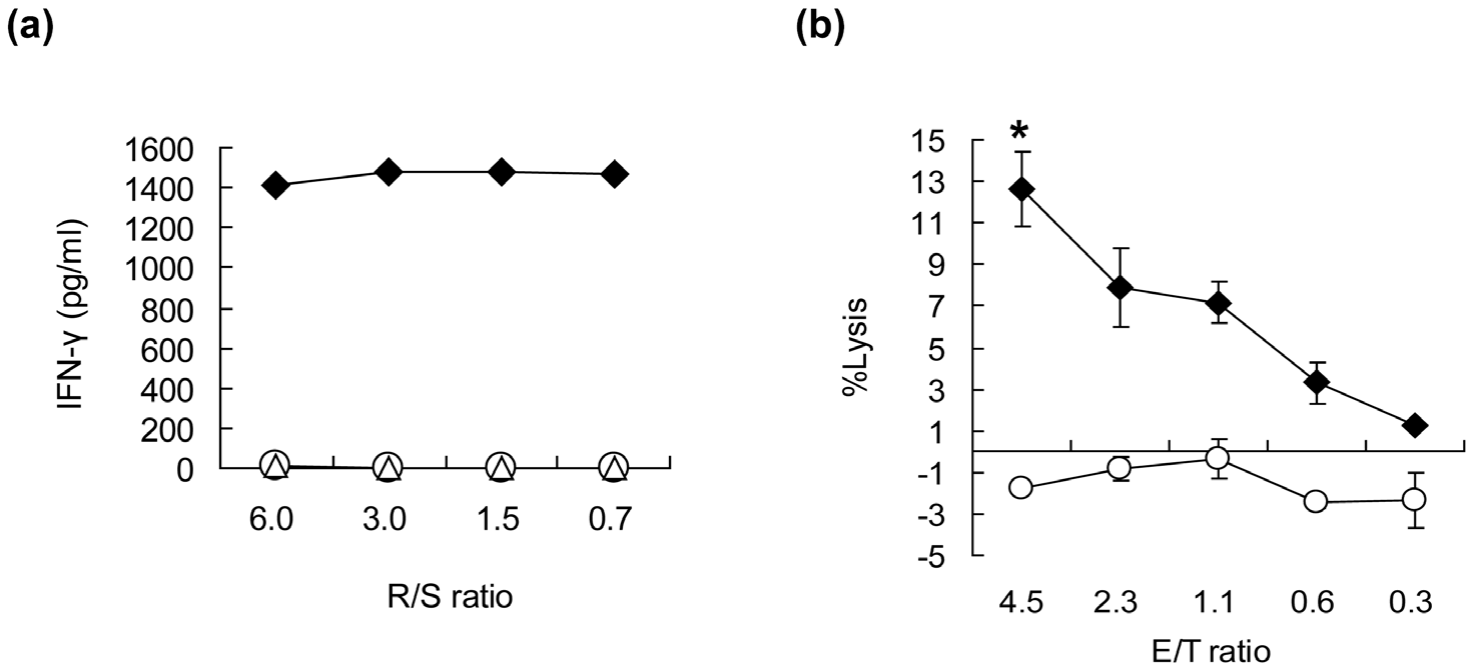
The recognition of HIG2 and HLA-A*02:01-expressing cells by a HIG2-9-4 peptide-specific CTL clone. (a) A HIG2-9-4 peptide-specific CTL clone was stimulated with COS7 cells expressing both HIG2 and HLA-A*02:01 (close diamond), or either HIG2 alone (open circle) or HLA-A*02:01 alone (open triangle), then the IFN-γ production was examined by ELISA. R/S ratio; responder/stimulator ratio. (b) The cytotoxic activity of the HIG2-9-4 peptide-specific CTL clone was examined against HLA-A*02:01-positive HIG2-expressing A498 cells (closed diamond) or HLA-A*02:01-negative HIG2-expressing Caki-1 cells (open circle). E/T ratio; effector/target ratio. All experiments were performed in triplicate. Representative results from three independent experiments are shown. *; *P*<0.001.

To further verify the recognition of HIG2-expressing cells with HLA-A*02:01 by the HIG2-9-4-specific CTL clone, we prepared COS7 cells in which either or both of two plasmids designed to express the full-length of HIG2 and HLA-A*02:01 were transfected. The HIG2-9-4-specific CTL clone produced IFN-γ when the cells were exposed to the COS7 cells expressing both HIG2 and HLA-A*02:01, while no IFN-γ production was observed when they were exposed to COS7 cells expressing either HIG2 or HLA-A*02:01 (Fig. 4a). Furthermore, the HIG2-9-4 peptide-specific CTL clone demonstrated cytotoxic activity against A498 cells expressing both HLA-A*02:01 and HIG2, while no cytotoxicity was observed against HIG2-expressing Caki-1 cells without HLA-A*02:01 expression (Fig. 4b).

### The HIG2-9-4 peptide cross-reacts with HLA-A*02:06

**Figure 5:**
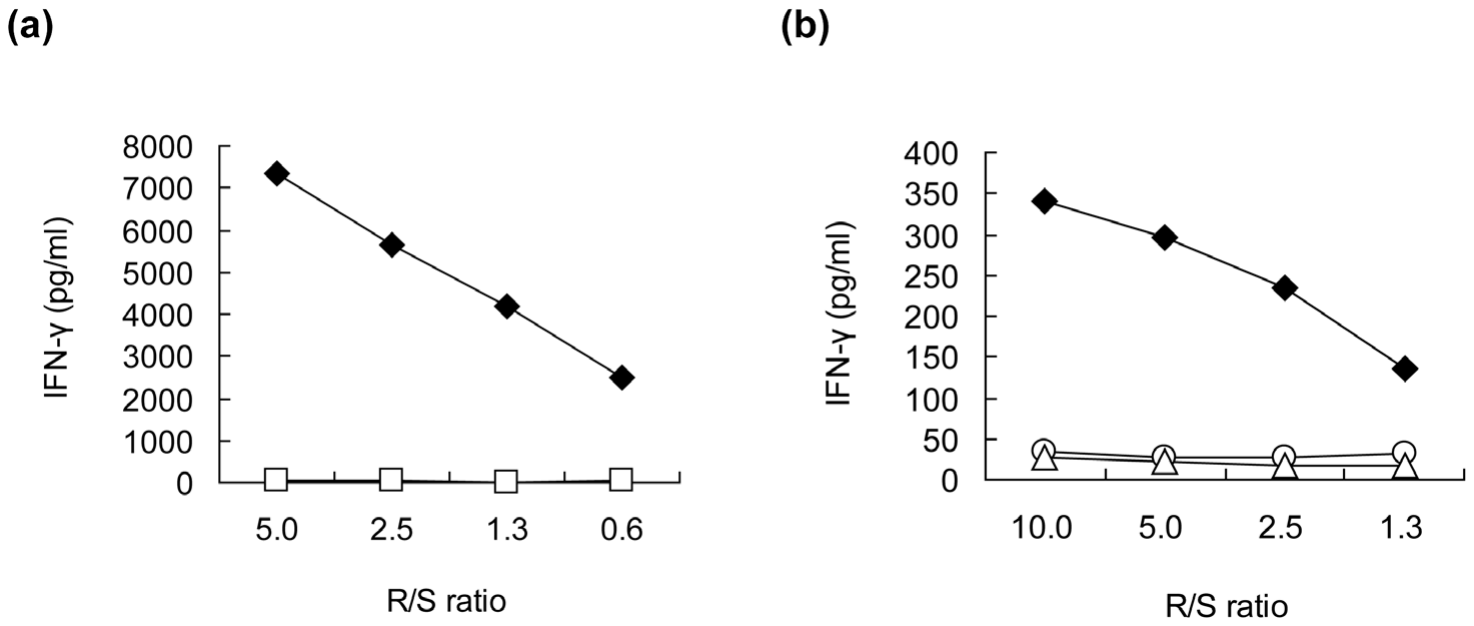
The HLA-A*02:06-restricted response of a HIG2-9-4 peptide-specific CTL clone. (a) A HIG2-9-4 peptide-specific CTL clone was induced from HLA-A*02:06-positive PBMCs, and stimulated with HLA-A*02:06-positive PSCCA0922 cells pulsed with the HIG2-9-4 peptide (close diamond) or HIV peptide (open square). (b) The HIG2-9-4 peptide-specific CTL clone was stimulated with COS7 cells expressing both HIG2 and HLA-A*02:06 (close diamond), or either HIG2 alone (open circle) or HLA-A*02:06 alone (open triangle). The IFN-γ production in the culture supernatant was examined by ELISA. R/S ratio; responder/stimulator ratio. The representative results from three independent experiments are shown.

We additionally evaluated the cross-reactivity of the HIG2-9-4 peptide with HLA-A*02:06, since HLA-A*02:06 differs from HLA-A*02:01 by a single amino acid, and some reports have indicated the presentation of HLA-A*02:01-restricted peptides on HLA-A*02:06 [33], [34]. Similar to the HLA-A*02:01 experiments, potent CTL clones were established from the PBMCs of HLA-A*02:06-positive donors by stimulation with the HIG2-9-4 peptide. An established CTL clone showed potent IFN-γ production when it was exposed to HIG2-9-4 peptide-pulsed HLA-A*02:06-positive PSCCA0922 cells (Fig. 5a). Furthermore, this CTL clone recognized COS7 cells that expressed both HIG2 and HLA-A*02:06 and produced IFN-γ, while no IFN-γ production was observed when stimulated with COS7 cells that expressed either HIG2 or HLA-A*02:06 (Fig. 5b). These results suggested that the HIG2-9-4 peptide is cross-reactive with HLA-A*02:06 to induce CTLs that show CTL activity against HLA-A*02:06- and HIG2-expressing cells.

## Discussion

The recent FDA approvals of the cellular immunotherapy, Sipuleucel-T (Provenge), and immunomodulatory antibody, ipilimumab (Yervoy), have provided a proof of concept that the immune system can be used as a new approach to treat cancer [35], [36]. Immunization with HLA-restricted epitope peptides derived from tumor antigens is a strategy that has been vigorously pursued to activate the immune system [37]-[40]. Unfortunately, many of the vaccine trials using epitope peptides failed to demonstrate clinical efficacy due, at least in part, to the potential immune escape mechanisms, which are attributed to the loss of tumor antigen expression by tumor cells [41]-[43]. Accordingly, the selection of tumor antigens which play a key role in tumor cell proliferation or survival is considered to be important to overcome immune escape. If a targeted tumor antigen is essential for tumor growth, the downregulation of this tumor antigen as a form of immune escape is expected to impair tumor progression.

Correspondingly, in the guidelines from the FDA (Guidance for Industry: Clinical Considerations for Therapeutic Cancer Vaccines), multi-antigen vaccines which contain multiple tumor antigens in order to generate multiple tumor-specific immunological responses were mentioned to effectively hinder escape mechanisms. We therefore consider that the identification of epitope peptides derived from multiple tumor antigens which are involved in tumor progression or survival can contribute to the development of multi-antigen vaccines, and can improve the efficacy of peptide vaccine therapies. We have previously identified epitope peptides derived from various tumor antigens, each of which plays a key role in tumor progression, and some of these peptides have been applied for clinical trials as multi-peptide vaccines [44]–[46].

In this study, we identified an HLA-A2 supertype-restricted epitope peptide derived from HIG2. HIG2 was upregulated in RCC and hardly detectable in normal organs except for the fetal kidney, and importantly, HIG2 expression was found to be directly associated with the proliferation of RCC cells [24]. Hence, RCC cells are thought to maintain HIG2 expression even under immunoselective pressure, or to otherwise exhibit tumor growth suppression resulting from the loss of HIG2 expression.

IFN-γ-producing stable CTL clones specific to the HIG2-9-4 peptide (VLNLYLLGV) were established from HLA-A2 (either A*02:01 or A*02:06)-positive PBMCs, and these clones responded specifically to COS7 cells that expressed both HIG2 and HLA-A2 (A*02:01 or A*02:06). We also revealed that HIG2-9-4-specific HLA-A*02:01-restricted CTLs exerted cytotoxic activity against RCC cells that were positive for both HIG2 and HLA-A*02:01, but not against negative cells. These results suggested that HLA-A2 (A*02:01 or A*02:06)-restricted HIG2-9-4 peptide-specific CTLs are inducible and stable, and these CTLs substantially respond to HIG2-expressing cells through the endogenous processing of the HIG2-9-4-peptide and the subsequent presentation with the HLA-A2 (A*02:01 or A*02:06) molecule on the cell surface. In addition, HIG2 is an oncofetal antigen, as described above, and no homologous sequence to the HIG2-9-4 peptide was demonstrated by a homology search using the BLAST algorithm. Thus, HIG2-9-4 peptide-specific CTLs should not induce unintended immunological responses to normal cells, such as those associated with autoimmune diseases, even if this novel and unique peptide induces strong immune responses against HIG2-expressing RCC.

HIG2 expression was found in the majority of RCC patients (86%) [25], and additionally, the HLA-A2 supertype is the most common HLA class I type in Caucasians and the second most common type in the Japanese population [26], [27]. Therefore, identification of HLA-A2 supertype-restricted epitope peptides derived from HIG2 could be applicable for immunotherapies in a wide variety of RCC patients. As well as finding novel tumor antigens which are widely expressed in cancer patients, finding epitope peptides restricted to major HLA Class I types will facilitate further development of cancer immunotherapies. We are now conducting clinical trials to examine the immunogenicity and safety of a HIG2-9-4 peptide vaccine in RCC patients.

## Supporting Information

Figure S1 **Response to the HIG2-9-8, HIG2-9-15, HIG2-9-4 or HIG2-10-8 peptide detected by IFN-γ ELISPOT assay.** The IFN-γ production from cells induced by the indicated peptide-pulsed DCs in 12 wells for each peptide was examined by an ELISPOT assay. “+” indicates the wells in which cells were stimulated with T2 cells pulsed with the indicated peptide and “−” indicates the wells in which cells were stimulated with HIV peptide-pulsed T2 cells. The wells in which the difference between peptide-pulsed cells and HIV peptide-pulsed cells were over 50 spots are indicated by squares.
(TIF)
