# Supplementary figures and images for: Identification of an HLA-A2-Restricted Epitope Peptide Derived from Hypoxia-Inducible Protein 2 (HIG2)
Source: PLoS One. 2014 Jan 8;9(1):e85267. doi: 10.1371/journal.pone.0085267 (PMC3885709; doi:10.1371/journal.pone.0085267)

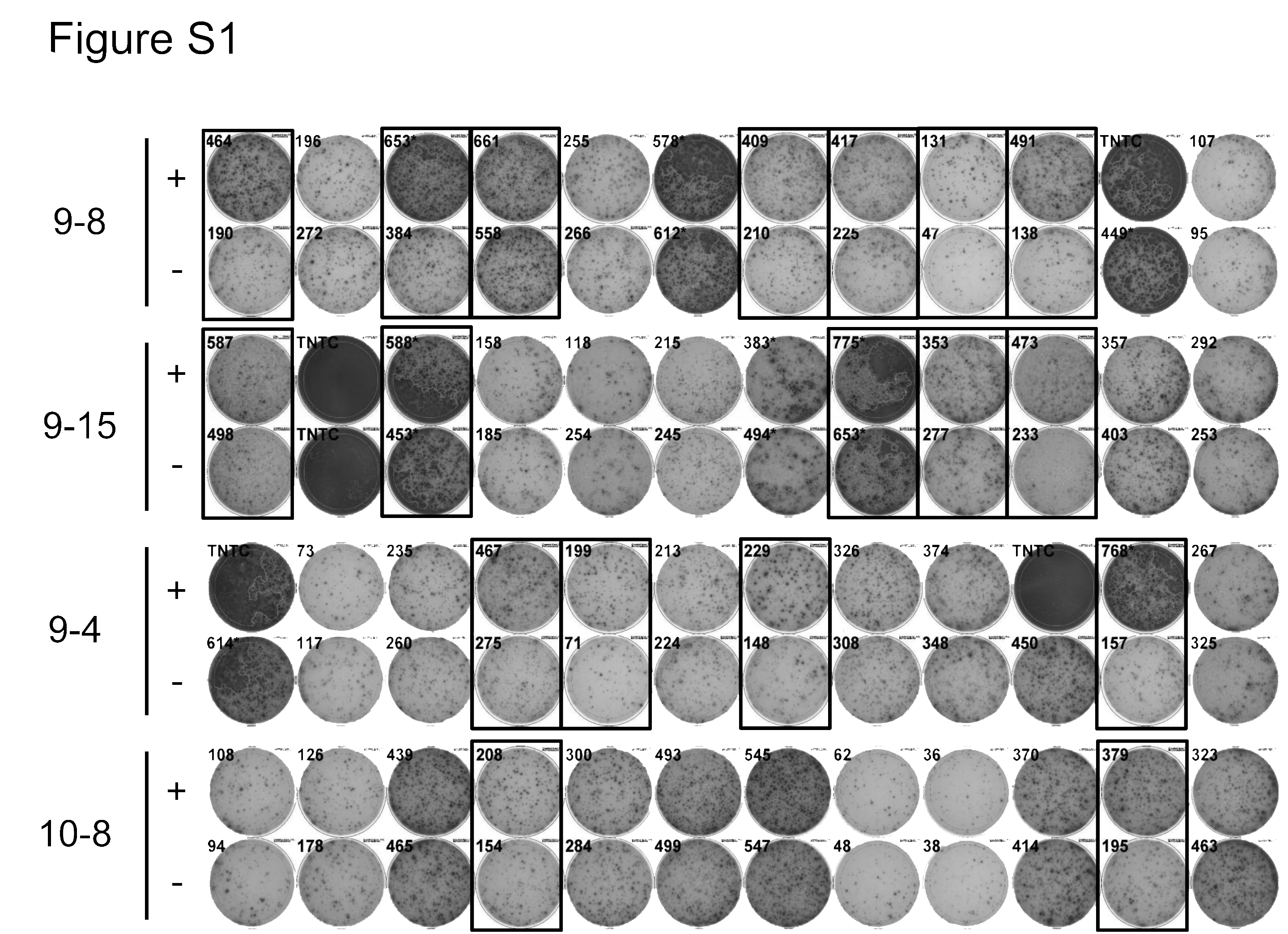

Supplement: Figure S1 — Response to the HIG2-9-8, HIG2-9-15, HIG2-9-4 or HIG2-10-8 peptide detected by IFN-γ ELISPOT assay. The IFN-γ production from cells induced by the indicated peptide-pulsed DCs in 12 wells for each peptide was examined by an ELISPOT assay. “+” indicates the wells in which cells were stimulated with T2 cells pulsed with the indicated peptide and “−” indicates the wells in which cells were stimulated with HIV peptide-pulsed T2 cells. The wells in which the difference between peptide-pulsed cells and HIV peptide-pulsed cells were over 50 spots are indicated by squares. (TIF) [file pone.0085267.s001.tif]
